# A reflection on modelling and examination of paramagnetic molecules for magnetic storage and molecular spintronics

**DOI:** 10.1039/d5sc90219c

**Published:** 2025-10-09

**Authors:** Mihail Atanasov, Shashank Vittal Rao, Frank Neese

**Affiliations:** a Max Planck Institut für Kohlenforschung Kaiser-Wilhelm Platz 1 D-45470 Mülheim an der Ruhr Germany mihail.atanasov@kofo.mpg.de neese@kofo.mpg.de rao@kofo.mpg.de; b Institute of General and Inorganic Chemistry, Bulgarian Academy of Sciences 1113 Sofia Bulgaria

## Abstract

Paramagnetic molecules featuring preferred orientation of their magnetic moments (magnetic anisotropy, single-molecule magnets, SMMs) are quite promising candidates for use in electronic devices like data storage and quantum computers (q-bits). In 2013, the authors published two papers on Fe^II^X_2_ with pseudolinear cores (J. M. Zadrozny, M. Atanasov, A. M. Bryan, C.-Y. Lin, B. D. Rekken, P. P. Power, F. Neese, and J. R. Long, *Chem. Sci.*, 2013, **4**, 125–138, https://doi.org/10.1039/C2SC20801F and M. Atanasov, J. M. Zadrozny, J. R. Long and F. Neese, *Chem. Sci.*, 2013,**4**, 139–156, https://doi.org/10.1039/C2SC21394J). Combining computational tools with *ab initio* ligand field theory, design principles have been formulated to afford predictions of SMM prior to their later synthesis. These efforts resulted in a linear Co^II^C_2_ SMM with magnetic anisotropy, the maximum possible for a 3d complex.

## Abstract 2

In celebration of our 15th anniversary and some of our most popular articles, Mihail Atanasov and Frank Neese reflect on the modelling and examination of paramagnetic molecules, building on two papers in Chemical Science: https://doi.org/10.1039/C2SC20801F & https://doi.org/10.1039/C2SC21394J.

## Graphical abstract

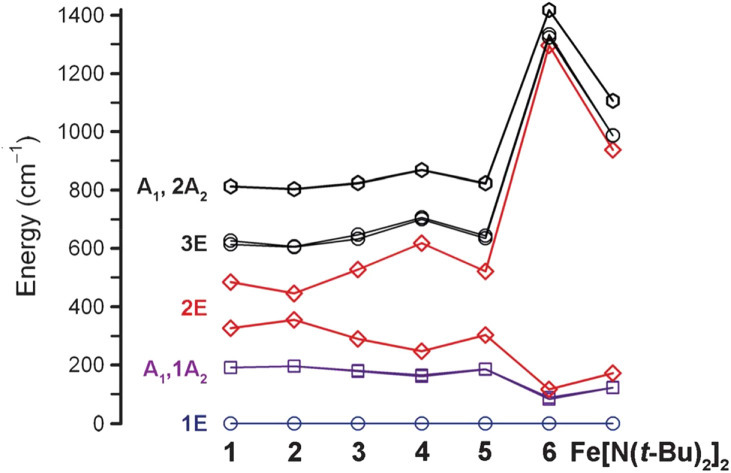

## Overview

**Fig. 1:**
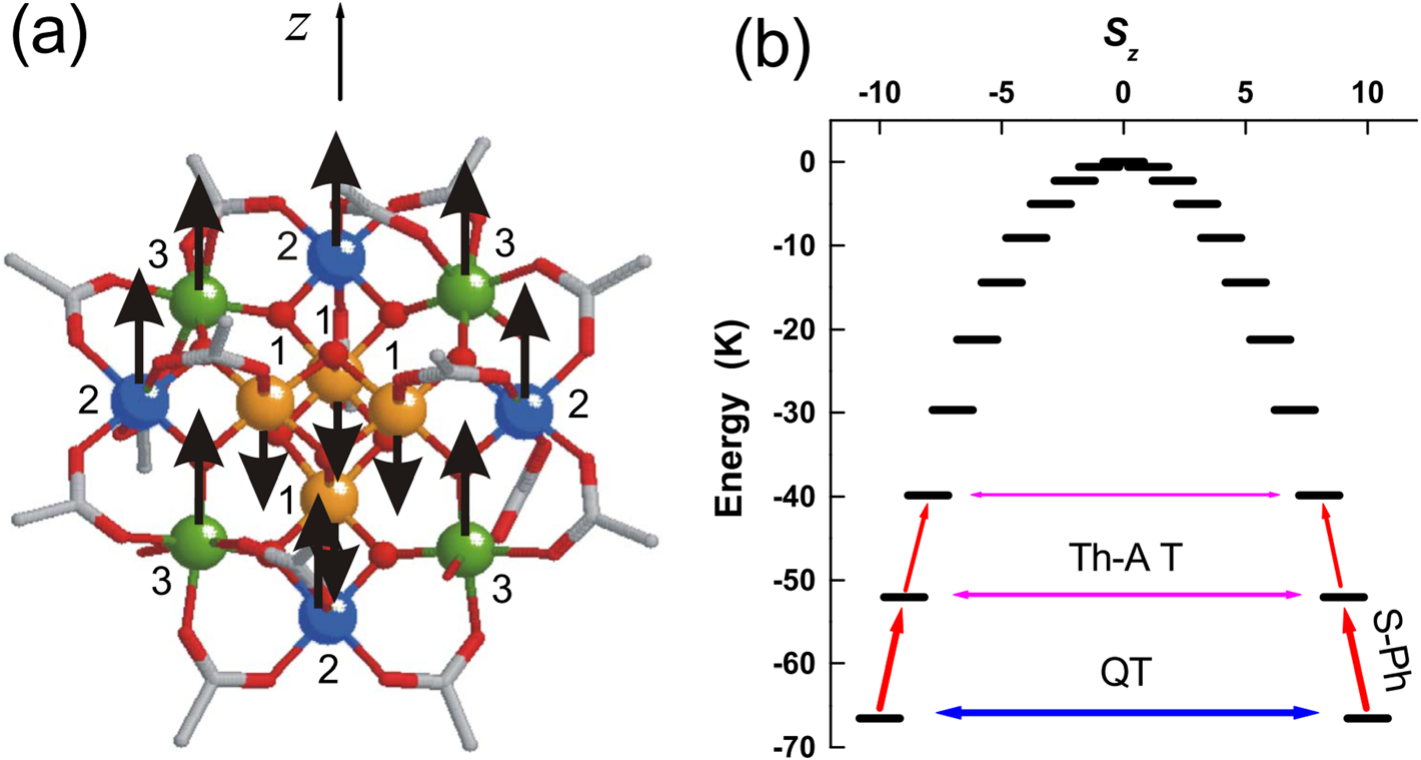
Structure of the Mn_12_O_12_(CH_3_COO)_16_H_2_O_4_ cluster^1^ showing the three crystallographically inequivalent Mn sites (a). Energy-level diagram for the magnetic pairs in zero field (b). Off-diagonal terms of the spin Hamiltonian allow transitions across the anisotropy barrier *via* quantum tunnelling (QT). Spin–phonon interactions (S–Ph) enable thermally assisted tunnelling (Th-AT) between excited doublets (this figure has been adapted from ref. 5 with permission of the American Physical Society, DOI: https://doi.org/10.1103/PhysRevB.76.184425).

Molecular magnetism aims at the design, synthesis, and characterisation of molecules that undergo spontaneous magnetization, similar to bulk magnets used for information processing and data storage. The field evolved after the discovery of a Mn_12_ cluster^1–3^ consisting of two subunits, one containing four Mn(iv) (d^3^) *S* = 3/2 ions sharing oxo bridges on the corners of a cube and coupling ferromagnetically to an *S* = 6 total spin, and another subunit of eight Mn(iii) (d^4^) *S* = 2 ions bridged by acetate and oxide anions, coupling ferromagnetically to an *S* = 16 total spin. The spins of the two subunits align antiparallel, resulting in an *S* = 10 ground state of the entire Mn_12_ cluster (Fig. 1a). This *S* = 10 ground state turned out highly anisotropic, implying a preferred orientation of the magnetic moment. The magnetic anisotropy stems from the close-to-parallel alignment of the local anisotropy axes of the eight *S* = 2 Jahn–Teller^4^ distorted Mn(iii) centres. An *E* = *DM*_S_^2^ (*M*_S_ = 10, 9, …, −9, −10) dependence of the energy of the ±*M*_S_ pairs with *D* = −0.5 cm^−1^ (zero-field splitting, ZFS) describes the ladder of the *M*_S_ spin manifold (Fig. 1b). When applying a magnetic field along the anisotropy axis *z*, the doubly degenerate ±*M*_S_ sublevels split pairwise, showing an energy *E*(−*M*_S_) < *E* (+*M*_S_) ordering. At temperatures lower than a given value, here *T*_B_ = 2 K (the blocking temperature *T*_B_), the state with the highest magnetic moment (*M*_S_ = −10) was shown experimentally to persist for two months. It loses its magnetisation upon an increase in temperature when sublevels with a different sign of *M*_S_ become equally populated, thus reaching thermal equilibrium.

## Discussion

**Fig. 2:**
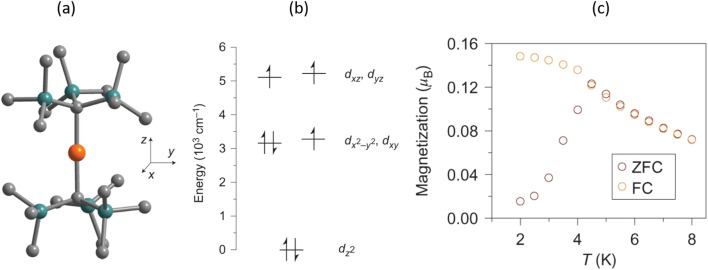
Structure of [Fe(C((SiMe_3_)_3_))_2_]^1−^ from the crystal structure of [K(crypt-222)][Fe(C((SiMe_3_)_3_))_2_]. Orange, turquoise and grey spheres represent Fe, Si and C, respectively (a). Energies of 3d molecular orbitals from *ab initio* ligand field CASSCF(NEVPT2) calculations (b). Low-temperature magnetisation data reveal blocking at 4.5 K, with divergence between field-cooled (FC) and zero-field-cooled (ZFC) measurements (c) (adapted from ref. 1 with permission of Nature Publishing Group, copyright 2013).

**Fig. 3:**
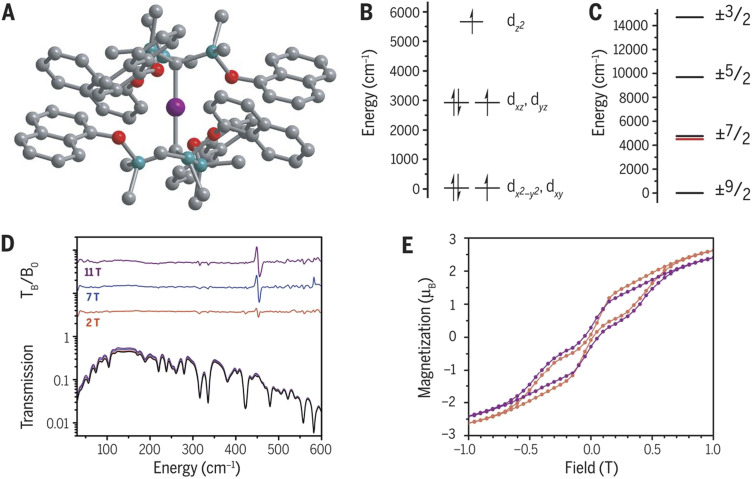
Linear dialkyl cobalt(ii) complex. (A) Molecular structure of Co(C(SiMe_2_ONaph)_3_)_2_. Purple, gray, turquoise, and red spheres represent Co, C, Si, and O, respectively. Hydrogen atoms have been omitted for clarity. (B) Energy diagram depicting the energy and electron occupations of the 3d orbitals. (C) The calculated splitting of the ground ^4^F state by spin–orbit coupling. The red line is the experimentally determined energy of the *M*_J_ = ±7/2 state. (D) Variable-field FIR spectra of Co(C(SiMe_2_ONaph)_3_)_2_. The top section shows the applied-field spectra (*T*_B_) divided by the zero-field spectrum (*T*_0_). (E) Variable-field magnetization data for pure Co(C(SiMe_2_ONaph)_3_)_2_ (orange) and diluted Co_0.02_Zn_0.98_(C(SiMe_2_ONaph)_3_)_2_ (magenta) at 1.8 K. *μ*_B_, Bohr magnetons (adapted from ref. 16, reproduced from M. Atanasov *et al.*, *Science*, https://doi.org/10.1126/science.aat7319).

Experimental efforts to increase the performance of this class of Mn_12_-type single-molecule magnets (SMMs) failed because of the weak dependence of the blocking temperatures on the number of spins (cluster size).^6^ To the extent that *T*_B_ is being governed by the energy quantity *E*_B_ = *DS*^2^ (over-barrier mechanism for the relaxation of the magnetization, Fig. 1b), and ignoring other spin-relaxation pathways (quantum tunnelling (QT) of magnetization and thermally assisted quantum tunnelling (Th-AT), see Fig. 1b), it was shown theoretically that because 
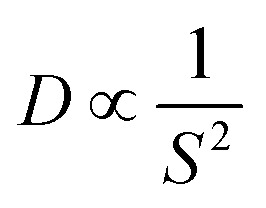
, *E*_B_ is only weakly dependent on the spin.^7,8^ This observation has led to a paradigm change in the field of SMM. Rather than aiming to increase the spin by increasing the number of Mn spin centres in the cluster, researchers turned their attention to the increase in magnetic anisotropy itself (the value of the negative *D*) focusing on complexes with one magnetic centre (single-ion magnets, SIMs). Magnetic anisotropy of such complexes arises from unquenched orbital moments, which maximize in linear complexes of Fe(ii) (d^6^), and Fe(i), Co(ii) (d^7^). In 2013, a collaborative team of experimentalists and theoreticians simultaneously published two papers (https://doi.org/10.1039/C2SC20801F, https://doi.org/10.1039/C2SC21394J).^9,10^ In ref. 9, FeX_2_ complexes of Fe(ii) that feature a d^6^ electron configuration with pseudo-linear FeO_2_, FeN_2_ and FeC_2_ cores were reported. Their magnetic anisotropy and slow relaxation of the magnetization have been analysed with respect to the nature of the ligands and the deviations from the linearity in terms of *ab initio* quantum chemistry calculations and ligand field theory (LFT). In ref. 10, we studied the effect of nuclear dynamics induced by the pseudo Renner–Teller effect^4^ on the magnetic anisotropy. Vibronic reductions in orbital contributions to net magnetic moments (and correspondingly the magnetic anisotropy *D*) were shown to exceed by far the orbital-moment quenching induced by metal–ligand covalency. Replacing C, N, or O donor atoms with their heavier analogues Si, P or S was shown experimentally^11^ and theoretically^12^ to increase spin–orbit coupling and therefore to mitigate vibronic coupling and thus enhance the magnetic anisotropy. It was further shown that avoiding secondary metal–ligand interactions *via* the use of sterically encumbered ligands with aliphatic rather than aromatic substituents has a favourable effect on the magnetic relaxation time.^13^ Using the same reasoning, it was predicted that bulky ligands of the former type are likely to support linear FeX_2_ cores and thus indirectly counteract unfavourable vibronic coupling.^10^ Expanding on these achievements and replacing the non-Kramer's Fe(ii) (d^6^) centre that has an integer total spin *S* = 2 with the Kramer's Fe(i) d^7^ ion that has a half-integer *S* = 3/2 spin, simultaneously reducing the ligand field strength and vibronic coupling, a complex with a linear Fe(i)X_2_ (1) core (X = C(SiMe_3_)^1−^) and magnetic blocking was first reported (Fig. 2a).^14^ The rather strong 3d_*z*^2^_–4s mixing, typical for low-valent first-row linear transition-metal complexes renders the 3d_*z*^2^_ orbital lowest in energy within the d-block. This leads to it being doubly occupied, thus supporting a ground state with one hole in the (3d_*xy*,*x*^2^−*y*^2^_)^3^ electronic configuration (Fig. 2b) and a nearly unquenched orbital-angular momentum *M*_L_ = ±2 that adds to the *S* = 3/2 spin magnetic moment of the ^4^*E* ground state. This resulted in an 
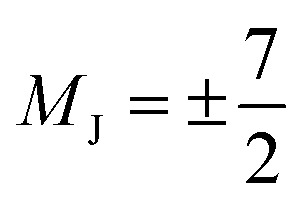
 relativistic ground state and Ising-type magnetic anisotropy. Magnetic blocking with a blocking temperature *T*_B_ = 4.5 K and molecular magnetic hysteresis were demonstrated.^14^ Being equipped with the predictive power of our computational tools (see below), we set out in 2015 to theoretically study the hypothetical analogue of (1) –[Co^II^(C(SiMe_3_)_3_))_2_] (2) and explored its spectroscopic and magnetic properties prior to it being synthesized later.^15^ The increase of the oxidation state by 1 from Fe(i) (d^7^) to Co(ii) (d^7^) in (2) and the switch of the position of the 3d_*z*^2^_ orbital from being the lowest in energy and doubly occupied in (1) to become the highest energy d-block orbital (and therefore singly occupied) in (2) resulted in two closely spaced sets of orbitals (d_*xy*,*x*^2^−*y*^2^_) < (d_*xz*,*yz*_) (Fig. 3B). A “non-Aufbau” occupation of each set being populated by three electrons was argued to reduce the overall inter-electronic repulsion, compared to the other alternative with a closed-shell d_xy_^2^d_x^2^−y^2^_ ^2^ and an open-shell (d_xz_^1^d_yz_ ^1^) configuration. The coexistence of the two open-shell configurations in the ^4^*E* ground state of (2) resulted in the maximally achievable orbital moment contributions to the total spin of *L* = 3, very much like the electrons in 4f (lanthanide) and 5f (actinide) orbitals that intrinsically feature an orbital angular moment of *L* = 3. A strongly anisotropic 
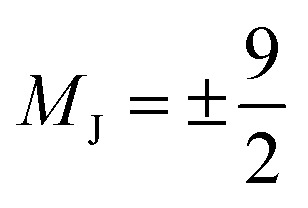
 (Fig. 3C) Ising-type relativistic magnetic ground state as the limit for the highest possible magnetic moment and magnetic anisotropy for 3d metal complexes was theoretically predicted. Fortunately, in 2018, the complex could be synthesized.^16^ As shown in Fig. 3A, the predicted structure, the barrier height for the relaxation of the magnetization (as revealed by field-dependent IR spectra, Fig. 3D), and the measured magnetic moments (Fig. 3D) are in excellent agreement with the theoretically predicted values.^16^ This study therefore demonstrated the power of computational tools when applied to the exploration of design principles of novel SMM prior to synthesis.

Computations of spectroscopic and magnetic properties of complexes of metals with open d- and f-shells have a long history in our group, rooted in numerous publications^17–22^ and development within the ORCA program project.^23^ All these efforts are available in the current version of ORCA, which, at the time of writing, is ORCA 6.1. In our work on magnetism, we have focused on the calculations of the spin-Hamiltonian (SH) parameters (**g** matrices, zero-field splitting tensors **D**, exchange couplings *J* and magnetic susceptibility curves), based on complete active space self consistent field (CASSCF) calculations^24^ with addition of dynamical correlation effects using N-electron valence perturbation theory to second order (NEVPT2),^25^ for spin-free energies and wave functions, and quasi-degenerate perturbation theory (QDPT)^17,18^ used in the calculations of spin–orbit multiplets. However, in cases of the (near) orbital degeneracies that are so characteristic of SIMs, the SH approach itself becomes invalid and in these cases, we have resorted to calculating physical observables directly from the relativistic wave functions obtained from the QDPT treatment. A third important ingredient of the methodology is *ab initio* ligand field theory (AILFT),^26^ allowing one to correlate magnetic parameters with the powerful language and culture of coordination chemistry.^27^ A final recent achievement is the MagRelax engine,^28^ available in the recent ORCA 6.1 release. It is based on pioneering contributions of Lunghi^29–31^ and Chilton^32,33^ for the calculation of magnetic relaxation times (or, equivalently, decay rates) *via* the spin–phonon coupling mechanisms.

## Outlook

We have shown here that quantum-chemical methods can play a key role in the rational design of single-molecular magnets prior to their synthesis. The use of *ab initio* methods for the prediction of magnetic relaxation times was already demonstrated.^29–33^ The diverse mechanisms of magnetic relaxation processes are currently actively under investigation. For instance, detailed computational methods are now available for the prediction of relaxation of magnetic states due to energy exchange between molecular and lattice vibration modes. Here, the processes can be one- or two-phonon driven. Moving forward, improvements in the quantitative precision of these methods would likely involve understanding the coherent spin transfer mechanisms, particularly in systems where coherent times are close in magnitude to magnetic relaxation time scales. This would involve solving the non-secular Redfield equations in greater detail^30,31^ to get the relevant rate matrices. Such results could be used and analysed applying multi-reference quantum chemistry methods. A long coherent spin-transfer time is of fundamental importance for the development of practical and usable spin systems for quantum computing and data storage.^34^ Moreover, for systems with a few relevant phonon modes, there is considerable value in building smaller, yet more accurate models that treat the spin-phonon coupling in an explicit quantum mechanical fashion, avoiding any statistical descriptions. These approaches have already shown promise in predicting phenomena such as avoided crossings in magnetic infrared (IR) and Raman^35^ spectra and long spin coherence times, as revealed by pulsed electron paramagnetic resonance (EPR) spectra of chelate V^IV^ (d^1^) complexes.^34^

## Author contributions

M. A., F. N. and S. V. R. wrote the manuscript.

## Conflicts of interest

There are no conflicts of interest to declare.

## Data Availability

The datasets used and/or analyzed during the current study are available from the corresponding author on reasonable request.
